# Effects of Body Mass Index and Full Body Kinematics on Tennis Serve Speed

**DOI:** 10.2478/hukin-2014-0003

**Published:** 2014-04-09

**Authors:** Francis KH Wong, Jackie HK Keung, Newman ML Lau, Douglas KS Ng, Joanne WY Chung, Daniel HK Chow

**Affiliations:** 1 Faculty of Health & Social Sciences, The Hong Kong Polytechnic University.; 2 Interdisciplinary Division of Biomedical Engineering, The Hong Kong Polytechnic University.; 3 School of Design, The Hong Kong Polytechnic University.; 4 School of Science and Technology, The Open University of Hong Kong.; 5 Department of Health & Physical Education, The Hong Kong Institute of Education.

**Keywords:** tennis serve, sport kinematics, ball speed

## Abstract

Effective training to improve serve speed is important for competitive tennis players. The purposes of this study were to investigate the effects of anthropometric factors and whole body kinematics of elite players on ball speed and to propose possible training strategies for improving the quality of tennis serves. Body and racket kinematics of tennis serves of 12 male elite Hong Kong players were investigated. The tennis serve was divided into four phases: I) Back-Swing Phase, II) Lead-Leg-Drive Phase, III) Forward-Swing Phase, and IV) Follow-Through Phase. It was shown that racket-side knee range of motion during phases II and III (r=0.705; p<0.05), racket-side knee peak extension velocity during phase II (r=0.751; p<0.01), racket-side hip peak extension velocity during phase II (r=0.657; p<0.05), racket-side shoulder range of motion in the coronal plane during phase III (r=0.616; p<0.05), racket-side elbow peak extension velocity during phase III (r=0.708; p<0.01) and body mass index (r=0.577; p<0.05) were significantly correlated with ball speed. Body mass index and the identified kinematic parameters that were significantly correlated with ball speed could be used as training guidelines for coaches and players to improve serve speed. Players should pay particular attention in training to increasing the extension velocity and range of motion of the identified joints.

## Introduction

Among the basic shots of tennis, the serve is used to start a point and advanced players try to produce a serve that cannot be returned by their opponent (Bahamonde, 2000; Elliott, 2001; Girard et al., 2005). Haake et al. (2003) showed that the number of good returns decreased and the number of aces increased as serve speed increased, especially for ball speeds over 161 km/h. Girard et al. (2005) reported that ball speeds over 200 km/h were regularly recorded in professional tennis matches. Ball speeds of lower-level tournament players were reported to range from 145 to 180 km/h (Girard et al., 2005).

The tennis serve is the only stroke in which a player has full control over the ball trajectory as a closed skill. However, it is difficult to master the serve as it involves complex coordination of the trunk, upper and lower limbs in a kinetic chain movement. Various kinematic and kinetic studies have been conducted to better understand the biomechanics of tennis serves by skilled players. Gordon and Dapena (2006) found that contributions to racket speed came sequentially from shoulder abduction, elbow extension, ulnar deviation rotation at the wrist, axial rotation of the upper trunk relative to the lower trunk, and wrist flexion. They also found that forearm pronation had a brief negative contribution to racket speed.

Elliott (1988) found that the linear velocities of various joints during tennis serves progressively increased from the knee, hip, shoulder, elbow and wrist and the summation of maximum resultant linear velocities of these joints produced the maximum angular velocity of the racket. In a subsequent study by Elliott et al. (1995), shoulder internal rotation was found to generate approximately 50% of linear racquet head velocity. Fleisig et al. (2003) studied the serve motion of players in the 2000 Olympics, quantifying the kinematics of players’ knees, pelvis, trunk, shoulders, elbows and wrists during high-velocity serves. They suggested that players should be trained to develop kinematic profiles similar to the 2000 Olympics players to produce effective high-velocity serves. However, the contribution of the ankle and hip segments to the kinematic chain of tennis serve has not been investigated (Fleisig et al., 2003; Elliott, 1988). As vertical drive from the legs is an important component of high speed tennis serves, whole body kinematics including analysis of both the ankle and hip segments should be investigated simultaneously for better understanding of the serve kinematic chain motion (Chu, 1996; Sweeney et al., 2012).

Given the importance of an accurate and high speed serve in winning matches, this study was conducted with the purpose of adding to existing data, racket and ball kinematics of tennis serves by including both ankle and hip segments in a full body analysis. The purposes of this study were to investigate the effects of anthropometric factors and whole body kinematics of elite players on ball speed and to propose possible training strategies for improving the quality of tennis serves. The results would help coaches and players to better understand the biomechanics of high speed tennis serves and formulate training strategies for improving the quality of tennis serves.

## Material and Methods

### Participants

Twelve male elite players with tournament experience were recruited from the Hong Kong tennis team. The mean (standard deviation) of their years of tennis training, training hours per month, age, body height, and body mass index (BMI) were as follows: 8.3 ±5.7 years, 36.9 ±39.9 hours, 20.5 ±3.8 years, 174.8 ±7.1 cm, and 22.2 ±2.8 kg/m^2^, respectively (Table 1). BMI was determined as the subject’s body mass divided by the square of his body height.

### Instrumentation

Body and racket kinematics of tennis serves performed by the players were captured using an 8-camera motion analysis system (Vicon Nexus MX, Oxford Metric, Oxford, UK) and correlated with peak ball speed measured using a radar speed gun (SOLO 2, Stalker, USA). Ball speed (km/h) was defined as the peak ball speed immediately after impact by the racket as measured by the radar gun, which has been commonly used in standard field measurement (Elliott et al., 2003; Fleisig et al., 2003; Girard et al., 2005). The radar gun was positioned at the baseline of the tennis court (approximately 23.7m from the net) and mounted on a 1m high tripod. It was set to point to the opposite side of the court directly and face the subject and the center of the racket at the point of ball contact. The radar gun was calibrated by the manufacturer with root-mean-square errors of 1.111, 1.246 and 1.906 km/h for strong, medium and weak signals, respectively.

In total, 43 retro-reflective markers were used, all 15 mm in diameter to define and measure 18 kinematic variables (Table 2). Thirty-nine markers (Table 3) were affixed to each participant to measure body kinematics and four markers were attached to the racket to register the instant of ball-racket impact. The marker system setup utilized followed Plug-in Gait Full Body Modeling of the Vicon motion capturing system (Vicon Nexus MX, Oxford Metric, Oxford, UK) previously used in gait analysis by Vaughan (1992). The coordinates of the markers were monitored by the motion analysis system with a sampling rate of 200 Hz (Chow et al., 2003; Elliott et al., 2003; Pappas et al., 1985). Joint motion was determined using the software provided by the manufacturer (Nexus software, Oxford Metrics Ltd, Oxford, UK). Angular displacements and velocities of the ankle, knee, hip, trunk, shoulder, elbow and wrist joints in the sagittal, frontal and the transverse planes were determined using the built in program of Vicon, Polygon by deriving ‘YXZ’ cardan angles from orientations of adjacent segments (Vicon Polygon, Oxford Metric, Oxford, UK). The data collected were filtered using the Woltring Filter program built into the system (Woltring, 1986). Validity and reliability of kinematic measurements were tested using MotionBuilder software (MotionBuilder 2009, Autodesk, USA).

### Protocol

The experiment was conducted at an indoor tennis court. All serves were performed with the participant’s leading foot adjacent to the centerline. Each participant performed a foot-up flat serve with minimum spin, so as to produce the fastest ball speed and this was assessed by the internationally qualified coach of the Hong Kong Professional Tennis Association. Separate individuals were responsible for reading and recording the radar gun results and calling the lines during the testing. Prior to the experiment, markers were affixed and each participant was allowed to take five practice trials. Brand new balls (Wilson, USA) were used by participants, but each used his own racket. Each participant was asked to serve at maximal speed until a total of seven successful serve trials (i.e. no service faults) were captured. Poor or invalid serves judged by the coach were excluded from the study. The best five successful serve trials landing in the service box with highest ball speeds were used for data analysis.

### Data Analysis

In analyzing body kinematics, we divided the tennis serve into four functional phases: I) Back Swing Phase (i.e. Preparatory Phase) defined from the instant of maximum shoulder internal rotation (MSIR) to the instant of maximum front knee flexion (MKF), II) Lead Leg Drive Phase defined from the instant of MKF to the instant of maximum shoulder external rotation (MSER), III) Forward Swing Phase defined from the instant of MSER to the instant of racket-ball impact (IMP); and IV) Follow-Through Phase defined from the instant of IMP to the instant of foot contact with the ground.

Data were analyzed using statistical software (IBM SPSS Statistics 20, IBM, USA) with the level of significance set at *p<0.05*. The intra-class correlation coefficient was used to confirm the reliability of the measurements. The Pearson’s coefficient of correlation was used to study the correlation between ball speed and the individual kinematic variables and demographic parameters (Tables 1 and 2). The subjects were also evenly divided into two groups by faster and slower serve speed and an independent samples t-test was used to compare the anthropometric and demographic variables between the two groups.

## Results

While age, training time and height did not have a significant correlation with ball speed, BMI was found to be significantly correlated (*r=0.577; p<0.05*) (Table 1). Divided into slower and faster serve groups, the 6 subjects with tennis serves under 150 km/h had a mean (±SD) BMI of 20.45 ±2.57 kg/m^2^, which was significantly smaller (*p=0.025*) than the 23.87 ±1.89 kg/m^2^ average BMI of the 6 subjects with tennis serves over 150 km/h.

In the kinematic analysis, it was found that ball speed was significantly and positively correlated with racket-side knee range of motion in the sagittal plane during phase II and III (*r=0.705; p<0.05*), racket-side knee peak extension velocity during phase II (*r=0.751; p<0.01*), racket-side hip peak extension velocity during phase II (*r=0.657; p<0.05*), racket-side shoulder range of motion in the coronal plane during phase III (*r=0.616; p<0.05*), and racket-side elbow peak extension velocity during phase III (*r=0.708; p<0.01*) (Table 2).

## Discussion

As the tennis serve is reported as the most difficult to master, yet most important stroke in the game, the study was conducted to investigate strategies for improving serves. In the current study, a deterministic model was adopted for identifying variables that could enhance ball serve speed (Elliott, 2006). Full body kinematics of Hong Kong elite tennis players during serve motion as well as peak ball speeds were captured. In addition to confirming the findings by Gordon and Dapena (2006) of sequence of joint motion during tennis serves, we demonstrated that greater ranges of motion and velocities of hip, knee, shoulder and elbow joints also contributed significantly to increased ball speed. In analysis of the lower limbs, it was found that the racket-side knee range of motion in the sagittal plane during lead leg drive phase and forward swing phase, racket-side knee peak extension velocity during lead leg drive phase, and racket-side hip peak extension velocity during lead leg drive phase were significantly and positively correlated with ball serve speed (Table 2). Chu (1996) reported that proper technique in serving involved more of a vertical drive with the legs than an arching and whipping action of the trunk. It has also been found that increased vertical linear velocity from the lower limbs increases vertical linear velocity drive of the racket-side shoulder leading to faster serves (Sweeney et al., 2012). Our findings concerning the positive correlation of increased knee range of motion, knee peak extension velocity, and hip extension velocity to greater serve speed may be due to contribution towards greater vertical drive.

The racket-side shoulder range of motion in the coronal plane during forward swing phase was found to be significantly correlated with ball serve speed in our subjects (Table 2). This indicated the importance of shoulder internal rotation in the serve action. These findings agreed with that reported by Elliott et al. (1995) that shoulder internal rotation generated approximately 50% of the linear racket head velocity. Fleisig et al. (2003) and Kibler (1995) also reported that high shoulder internal rotation velocity measurement was critical for producing high ball serve velocity.

A study, by Gordon and Dapena (2006) found the contributions to the speed of the racket came sequentially from shoulder abduction, elbow extension, ulnar deviation rotation at the wrist, twist rotation of the upper trunk relative to the lower trunk, and wrist flexion. In the current study, racket-side elbow peak extension velocity during forward swing phase was found to be significantly correlated with ball serve speed (Table 1). However, Sprigings et al. (1994) reported that elbow extension at contact was counter-productive to racket head speed and Elliott et al. (1995) as well as Sprigings et al. (1994) noted that elbow extension played a negative role and reduced the forward velocity of the center of the racket at impact. Although elbow extension at the instant of ball impact was shown to be counterproductive in previous studies, we found that greater racket-side elbow peak extension velocity could result in faster serve speeds.

Significant association between body height and serve speed was reported by Vaverka and Cernosek (2013). Although a positive correlation was also found in our study, it was not statistically significant. This might be due to the small size used in the current study. It was interesting to find that BMI was significantly correlated with ball serve speed. No previous study has reported a correlation between BMI and serve speed. It was suggested by Signorile et al. (2005) that anthropometric data may further improve the predictive ability of serve velocity equation. The correlation may also be explained by Allometry theory (Wrigley, 2000) that body mass was related to torque production and thus, an increase in body mass in relation to body size would increase torque, which in turn would increase serve speed. It may be important to note that national team players with higher BMI are likely to have higher lean muscle mass. Therefore, in addition to improving body kinematics during serves to match those of faster serving players, increasing BMI by increasing muscle mass may also improve serve speed both by increasing power and torque production. Results of the present study may be used as evidence for guidelines of monitoring BMI in tennis players.

In comparing the kinematics of elite tennis players, there were significant differences in the kinematic pattern between faster and slower serves. Fleisig et al. (2003) investigated the kinematics used by world class tennis players in producing high-velocity serves and suggested that coaches worldwide should take note of the differences between the profiles in training their players so as to achieve high-speed serves. In order to learn the advanced skills and training exercises of elite players, training courses should be provided for coaches and athletes focusing on movement specifics such as joint angles and velocities. In particular, the results suggest that it may be beneficial to focus on training knee, hip and elbow extension for higher peak velocities. This is thought to improve serve speed by increasing vertical drive of the legs and upward motion of the racket. In addition, as range of motion was correlated to ball speed, flexibility training may be advantageous for athletes who are unable to reach ideal ranges of motion, particularly at the shoulder joint. Motion analysis could be used to assess the flow of energy across segments and the movement pattern and provide training indicators directly to tennis players. Analysis of the player kinematic profile and ball serve speed could be used as references for setting up a scientific training protocol for elite players. While skilled players may have mastered the basics of serving, many may need to fine-tune their movement for further improvement. As movement is also affected by body composition, following BMI guidelines may be beneficial.

Although tennis kinematics can be applied worldwide, only local Hong Kong players were used in this study. The current Hong Kong Tennis Team consists of 15 members and they were all invited to take part in the research, however, due to time conflicts, only 12 tennis players participated in the study. The number of subjects was comparable with the studies by Elliott et al. (2003) and Reid et al. (2008).

## Conclusions

In our full body kinematic analysis, ranges of motion and velocities of particular joints were identified to be significantly related to serve speed. These included racket-side knee and shoulder range of motion along with peak extension velocity of the racket-side knee, hip and elbow. In addition, as greater BMI was found to be associated with faster serve speed, an increase of BMI by increasing muscle mass may improve serve speed both by increasing power and torque production.

Serve kinematics could be adjusted to improve serve ball speed and may allow players to improve towards a world class level in terms of serving. Longitudinal studies are needed to investigate the relationships between various serve kinematics as they develop over time in younger players. Comparison of variation of serve kinematics and ball speed after intervention can be a possible area for future research.

## Figures and Tables

**Table 1:** Anthropometric and Demographic Correlates with Serve Speed

	**Mean**	**SD**	**Min**	**Max**	**r value**
Age (years)	20.5	3.8	13.0	25.0	0.526
Body Height (cm)	174.8	7.1	170.0	184.5	0.542
Body Mass Index (BMI) (kg/m^2^)	22.2	2.8	16.5	27.5	0.577^*^
Years of Tennis Training	8.3	5.7	2.0	19.0	0.556
Training Hours per Month	36.9	39.9	10.0	150.0	0.090

* Significance *p<0.05*

**Table 2:** Correlates of Serve Kinematics and Ball Speed

**Movement**	**r value**
Racket-side ankle range of motion in the sagittal plane during phase I and II (°)	−0.275
Racket-side ankle peak plantar flexion velocity in the sagittal plane during phase II (°/s)	−0.232
Racket-side rear knee range of motion in the sagittal plane during phase II and III (°)	0.705^*^
Racket-side rear knee peak extension velocity during phase II (°/s)	0.751^**^
Racket-side rear hip range of motion in the sagittal plane during phase I and II (°)	0.505
Racket-side rear hip peak extension velocity in the sagittal plane during phase II (°/s)	0.657^*^
Racket-side pelvic rotation range of motion in the coronal plane during phase I to III (°)	−0.128
Pelvic peak axial rotation velocity during phase II (°/s)	−0.210
Trunk range of motion in the coronal plane during phase I to III (°)	−0.095
Racket-side trunk peak rotation velocity during phase II (°/s)	0.201
Racket-side shoulder range of motion in the coronal plane during phase III (°)	0.616^*^
Racket-side shoulder peak internal rotation velocity in the horizontal plane during phase III (°/s)	0.212
Racket-side elbow range of motion in the sagittal plane during phase II and III (°)	0.435
Racket-side elbow peak extension velocity during phase III (°/s)	0.708^**^
Racket-side wrist range of motion in the coronal plane during phase III (°)	−0.005
Racket-side wrist peak pronation velocity during phase III (°/s)	0.260
Racket-side wrist range of motion in the sagittal plane during phase III (°)	−0.154
Racket-side wrist peak flexion velocity during phase III (°/s)	0.432

Phases I, II, III & IV denoted the Back Swing Phase, Lead Leg Drive Phase, Forward Swing Phase and Follow-Through Phase respectively.

* Significance p<0.05;

** Significance p<0.01

**Table 3:** Anatomical Landmarks for Marker Placement

**No.**	**Marker**	**Definitions**
1 & 2	L/R front head	Approximately over the bilateral temples
3 & 4	L/R back head	Posterolateral of head at the level of the front head markers
5	C7	Spinous process of the 7th cervical vertebra
6	T10	Spinous Process of the 10th thoracic vertebra
7	Right back	Middle of the right scapula (for side identification)
8	Clavicle	Jugular notch
9	Sternum	Xiphoid process of the sternum
10 & 11	L/R ASIS	Bilateral anterior superior iliac spine
12 & 13	L/R PSIS	Bilateral posterior superior iliac spine
14 & 15	L/R shoulder	Bilateral acromio-clavicular joint
16 & 17	L/R upper arm	Bilateral upper arm between the left elbow and left shoulder
18 & 19	L/R elbow	Bilateral lateral epicondyle
20 & 21	L/R forearm	Bilateral lower arm between the wrist and elbow markers
22 & 23	L/R wrist thumb	Bilateral wrist thumb side
24 & 25	L/R wrist ulnar	Bilateral wrist ulnar side
26 & 27	L/R fingers	Bilateral dorsum of the hand below the head of the 2nd metacarpal
28 & 29	L/R thigh	Lower lateral 1/3 surface of the bilateral thigh
30 & 31	L/R knee	Lateral epicondyle of the bilateral knee
32 & 33	L/R tibial wand	Lower 1/3 of the bilateral shank
34 & 35	L/R ankle	Bilateral lateral malleolus
36 & 37	L/R heel	Bilateral calcaneous at the same height of the toe marker
38 & 39	L/R toe	Bilateral 2nd metatarsal head

L/R = Left & Right
